# Unveiling the enterovirus diversity in Barcelona, Spain (2020–2024) through wastewater and clinical surveillance

**DOI:** 10.1080/22221751.2025.2589547

**Published:** 2025-11-25

**Authors:** David Garcia-Pedemonte, Albert Carcereny, Cristina Andrés, Andrés Antón, Ian Pérez, Albert Blanco, Cristina Fuentes, Maria Isabel Costafreda, Patricia Nadal-Barón, Belen Galofré, Miquel Paraira, Jacobo Mendioroz, Rosa M. Pintó, Albert Bosch, Susana Guix

**Affiliations:** a Enteric Virus Laboratory, Department of Genetics, Microbiology and Statistics, School of Biology, Research Institute of Nutrition and Food Safety (INSA-UB), University of Barcelona, Barcelona, Spain; b Respiratory Viruses Unit, Microbiology Department, Vall d’Hebron Hospital Universitari, Vall d’Hebron Institut de Recerca (VHIR), Vall d’Hebron Barcelona Hospital Campus, Barcelona, Spain; c Biomedical Research Networking Center in Infectious Diseases, Instituto de Salud Carlos III (ISCIII), Madrid, Spain; d Aigües de Barcelona, Barcelona, Spain; e Public Health Office, Health Departament, Generalitat de Catalunya, Barcelona, Spain

**Keywords:** Enterovirus, wastewater-based epidemiology, clinical surveillance, environmental surveillance, public health

## Abstract

Enterovirus (EV) infections are common and often mild or asymptomatic but can occasionally cause serious illness in children. Routine clinical surveillance typically underestimates the full spectrum of circulating EVs, whereas wastewater-based epidemiology captures a broader viral diversity and may serve as an early warning tool. We conducted a 5-year surveillance (2020–2024) in Barcelona, Spain, combining biweekly wastewater sampling with clinical EV reporting during the SARS-CoV-2 pandemic and subsequent relaxation of restrictions. We quantified EV genome copies in sewage by RT-qPCR and performed partial VP1 sequencing on wastewater concentrates and on clinical specimens. Over the study period, wastewater EV loads broadly paralleled reported case trends, with good correlations over the years except in 2022. A monotonic relationship was observed for certain EV type trends, including CVA6, CVB2, CVB3, E11, and EV-D68, but there was low robustness in their predictive capacity for clinical cases. Overall, deep sequencing revealed 122 distinct EV types in wastewater versus 92 types in clinical samples; 66 types were found in both sources, whereas 56 types appeared only in sewage. Sequences from wastewater and clinical sources showed high genetic similarity. Dominant EV types shifted over time highlighting the virus dynamics. These findings demonstrate that integrating WS with clinical surveillance yields a more comprehensive picture of EV circulation, uncovering hidden viral diversity and enabling early detection of emerging public health threats.

## Introduction

Following the International Committee for the Taxonomy of Viruses (ICTV) [1], genus *Enterovirus* comprises seven species infecting humans: *Enterovirus alphacoxsackie* (formerly *Enterovirus A*)*, Enterovirus betacoxsackie* (*Enterovirus B*), *Enterovirus coxsackiepol* (*Enterovirus C*)*, Enterovirus deconjuncti* (*Enterovirus D*)*, Enterovirus alpharhino* (*Rhinovirus A*)*, Enterovirus betarhino* (*Rhinovirus B*) *and Enterovirus cerhino* (*Rhinovirus C*). However, the former nomenclature will be used throughout the manuscript.

Although human enterovirus (EV) infections are often mild or asymptomatic, they may cause severe diseases like encephalitis, meningitis, myocarditis, poliomyelitis, and acute heart failure [2–4]. These viruses are transmitted via the faecal-oral or the respiratory routes. Even though EV infections can occur at any age, young age groups (<5 years old) tend to be more susceptible than adults, thus potentially showing more acute symptoms [5]. Moreover, infection with a specific enterovirus serotype typically induces long-lasting, type-specific immunity, but does not confer cross-protection against other serotypes.

Poliovirus (PV) is the most well-known EV associated with acute flaccid paralysis (AFP) [6]. Despite being on the verge of eradication, circulating vaccine-derived poliovirus type 2 (cVDPV-2) has been detected through wastewater surveillance in Europe, first in Barcelona in Sept-2024, and later detected in Poland, Germany, Finland, and the United Kingdom [7]. Thankfully, the high inactivated vaccine coverage in Europe prevented the occurrence of AFP cases. However, other EVs such as EV-A71 and EV-D68 may also be related to AFP, thus highlighting the importance of a comprehensive monitoring of non-polio EVs [8–10].

Since the SARS-CoV-2 pandemic, wastewater surveillance (WS) has been widely used to monitor the circulation of different viruses in the community, providing information on total infections regardless of symptoms occurrence, and providing complementary information to clinical data, particularly when such data are limited or unavailable [11,12]. In recent years, WS studies based on amplicon next generation sequencing (NGS) have been used to elucidate the diversity of circulating EVs [13–16]. In this study, we aim to monitor EV genome copy (GC) levels and diversity in sewage from the metropolitan area of Barcelona (covering around 2.7 million inhabitants) over a 5-year timespan (2020–2024) and compare the results with clinical data from the same timeframe.

## Materials and methods

### Wastewater sampling, concentration, and extraction

The Besòs (BSS) and Prat de Llobregat (PDL) wastewater treatment plants (WWTP) in the metropolitan area of Barcelona, covering a population of 1.8 and 1.1 million inhabitants, respectively, were biweekly sampled from Jul-2020 to Dec-2024. At BSS, from Jul-2020 to Jun-2024, grab samples (1L) were collected at the time of maximum faecal excretion, monitored through fecal coliform determination. From Jul-2024 to Dec-2024, 24h-composite samples (1L) were collected instead. At PDL, 24h-composite samples were collected throughout the study, except between Apr-2023 and Jul-2024, when grab samples were used. Samples were transported at 0–4°C and processed immediately or stored at 4°C upon arrival and processed the following day.

Viral particles were concentrated from 200 mL of raw wastewater using a previously described adsorption-precipitation method based on aluminium hydroxide [17]. From Apr-2023 onwards, a centrifugation (4000 x g, 10 min) before the concentration process was performed to eliminate debris and inhibitors. As process control, samples were spiked with 1.86 × 10^6^ TCID_50_ units of vMCo Mengovirus strain [18] before the concentration step. Viral recovery was estimated using an RT-qPCR [18] and recovery rates ranged between 27% and 93%, with an average of 55% ± 18%.

RNA extraction was performed from 150 µL of concentrate using the Maxwell® RSC PureFood GMO and Authentication Kit (Promega) following the manufacturer’s instructions.

### EV quantification and typing in wastewater

An in-house RT-qPCR targeting a 146 nt conserved region of the 5’ end of the genome was used to detect and quantify EV in wastewater [19]. The calculated limit of detection was 4 GC/reaction, and the limit of quantification was 8.5 GC/reaction. Reactions were performed in CFX96 BioRad or QuantStudio 3 (Thermo Fisher Scientific) instruments. Viral concentrations were expressed as genome copies per litre (GC/L) and normalized by the daily sewage flow rate (L).

For typing, a pan-EV protocol based on a 348–393 bp partial amplification of the VP1 gene [20] was performed once a month. The PCR product was purified with magnetic beads (Beckman Coulter, Brea, CA, USA) using a 1:1 ratio and quantified with Qubit® dsDNA HS Assay Kit (Thermo Fisher Scientific, Waltham, MA, USA). Deep sequencing was performed using the MinION platform (Oxford Nanopore, UK). Samples obtained prior to the end of Mar-2023 were sequenced using R9.4.1 flow cells (FLO-MIN106D) and V12 reagents following the Nanopore Classic PCR tiling of the SARS-CoV-2 virus protocol [21], which could be adapted for EV sequencing as the amplicon size was very similar. For samples obtained from April 2023 onwards, R10.4.1 flow cells (FLO-MIN114) and V14 reagents were used, and the same sequencing protocol was followed. MinKNOW software was used for sequencing run setting, data collection and both basecalling and demultiplexing. The software was regularly updated to ensure access to all released enhancements.

Raw FASTQ files were processed with an approach based on the VSEARCH tool [19,22]. Briefly, VSEARCH v2.28.1 was used to (i) trim 30 nucleotides at both ends of the reads, (ii) discard sequences shorter than 250 bp and longer than 600 bp, (iii) generate clusters at 95% identity and (iv) perform a BLAST search with a minimum id of 80% against an in-house EV database containing 6396 sequences (downloaded from NCBI) comprising human and non-human EV. EV proportions were automatically calculated and plotted for each processed sample.

Both EV concentration and typing results were plotted using R v4.4.1 software with ggplot2 package.

To estimate both the amount and types of circulating EV in the Barcelona area, a mean value was calculated using BSS and PDL results.

### Human sampling, EV quantification and typing

Specimens from patients presenting symptoms suggestive of an EV-related acute respiratory or neurological illness were received in the laboratory for virological diagnosis of the Vall d’Hebron Hospital, the largest hospital in Barcelona, which performs EV diagnosis and typing of samples derived from all primary care centres within the Barcelona metropolitan area. Only sequences from patients from the same area covered by the WWTPs were selected for further analysis. Similar to previous studies from a hospital setting, the enterovirus detection was performed by commercial real-time multiplex RT–PCR assays [23] (Allplex Respiratory Panels 1A-3 for respiratory samples or Allplex Meningitis-V2 Assay for others, Seegene, South Korea), while the genetic characterization of those EV-laboratory-confirmed cases was carried out by phylogenetic analysis of the partial viral protein 1 (VP1) coding-region according to the protocol from the World Health Organisation, with minor modifications [20].

### Correlation analysis

Correlation analysis between wastewater EV levels and the total clinical cases reported during a period of two weeks before and after the collection date, or on the same day as the wastewater collection date was performed using Spearman. Similarly, to evaluate monotonic relationships, Spearman correlation coefficients were calculated between each EV type levels in sewage, estimated multiplying the total EV GC determined via RT-qPCR and the proportion of each EV type determined via NGS, and the corresponding number of clinical cases reported two weeks after the sewage sampling date. Additionally, we fitted a quadratic regression model where wastewater GC were used to predict 14-day accumulated clinical cases, and the coefficient of determination (*R*^2^) was calculated as an estimate of the proportion of variance in clinical cases explained by wastewater signal. Autocorrelation in the time series data was assessed using the Box–Ljung test with a lag of up to 14 days, performed in R (v4.5.1). GraphPad Prism v10.2.3 was used to calculate the correlations and their significance. Significant correlations in the ranges 0.7–1, 0.3–0.7 and <0.3 were considered strong, fair and poor respectively.

### Phylogenetic analysis

Clustered sequences (95% identity) were obtained as described above and used for the phylogenetic analysis. For each EV type found in each wastewater sample, the consensus sequence from the cluster with the highest number of sequences and a second one randomly selected were used to build the phylogenetic tree. For clinical samples, one consensus sequence per sample was included, as no intra-sample EV population diversity was expected. Additionally, reference sequences (NCBI) for each found EV type were included. A multiple sequence alignment (MSA) was performed with MAFFT v7.526 using the L-INS-I model. IQ-TREE v.2.3.4 was used to find the best-fitting model tree. FigTree v1.4.5 was used for tree visualization and colouring.

### Amino acid mutation analysis

Cluster sequences were translated, and identical ones merged. Clinical sequences were also translated and identical sequences merged. MMseqs v.15.6f452 was used to filter and align the wastewater sequences against the clinical sequences. Wastewater amino acid sequences with a sequence identity lower than 95% and with less than 90% coverage compared to the clinical sequences were discarded. Sequences passing these filters were used for mutation analysis. Mutations found in wastewater sequences with frequencies over 10% were reported.

## Results

### EV quantification in Barcelona sewage and correlation with clinical cases

**Figure 1.**
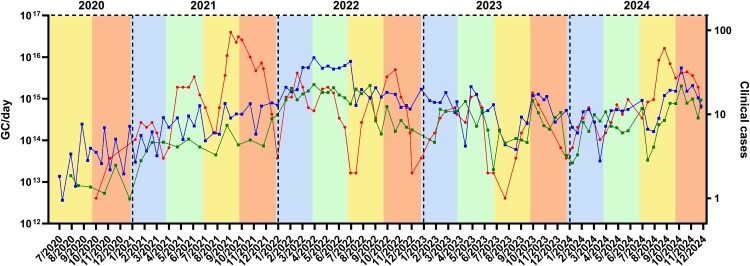
Enterovirus levels in sewage and clinical cases reported in Barcelona during 2020–2024. Blue and green squares represent the EV GC per day detected in wastewater by RT-qPCR in the BSS and PDL WWTPs, respectively. Red circles indicate the reported number of clinical cases two weeks after each date. Both datasets are represented in logarithmic scale. Background colours mark each season of the year; blue – winter, green – spring, yellow – summer and orange – autumn. Vertical black dashed lines mark the end of each year.

All sewage samples tested positive for EV. As shown in Figure 1, GC levels from BSS WWTP were consistently higher than those from PDL WWTP, and showed a similar seasonal trend. Highest concentrations were seen in spring-summer 2022 and autumn 2024. From 2020 on, GC progressively increased and peaked to maximum numbers during spring 2022. Similar seasonal trends were observed during 2023 and 2024 with viral loads decreasing in the summer periods (Figure 1). Aggregated clinical cases from two weeks after each wastewater sampling date showed a similar pattern with two clear peaks during spring and early autumn (Figure 1).

Spearman’s correlations between sewage EV levels and clinical cases aggregated from 2 weeks after wastewater sampling date showed a fair correlation value for the total 5-year period (ρ = 0.46, *p* < 0.001); for the individual years, the correlations were generally stronger, except for 2022 (Figure S1). Correlations between sewage levels and clinical cases reported before or on the sampling date showed a similar trend (Figure S1).

Correlation analysis also confirmed a similar trend in both WWTPs despite differences in the sampling method (grab versus composite). Spearman correlation value between viral loads from both WWTPs throughout the total study period showed a strong correlation (ρ = 0.75, *p* < 0.001). A similar correlation was observed from Jul-2020 to Apr-2023, when grab and composite samples were collected from BSS and PDL WWTPs, respectively (ρ = 0.82, *p* < 0.001), suggesting that the use of different sampling methods was not introducing significant bias to the results.

### EV diversity in Barcelona sewage

**Figure 2.**
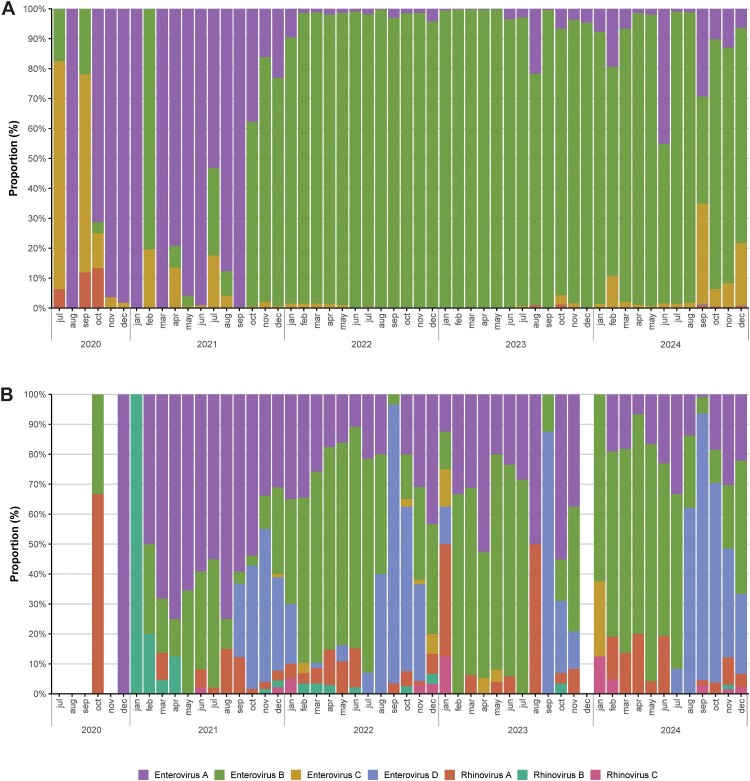
Relative abundance of EV species in Barcelona during 2020–2024. Monthly EV sequencing data from (A) wastewater samples, where each bar represents the mean percentage from both Barcelona WWTPs (PDL and BSS), and (B) reported clinical cases associated with medical assistance.

During the 5-year period of the study, a total of 6.49 × 10^7^ raw reads were obtained from wastewater samples, of which 4.52 × 10^7^ (70%) passed the filters and matched with human infecting EV types. The median sequencing depth was 184,639 reads (from 268 to 2,239,093 reads) with only 8 samples having less than 1000 reads. EV levels of sequenced samples ranged between 1.19 × 10^4^ and 1.37 × 10^7^ GC/L (mean 2.37 × 10^6^ GC/L) and the lowest sequencing output was obtained when the EV viral load was of 1.19 × 10^4^ GC/L. EV-B was the most dominant throughout the surveillance, except from Jul-Sep-2020 and Oct-2020 to Sep-2021 when EV-C and EV-A were respectively found in higher proportions. EV-D and rhinovirus (RV) A, B and C were found in lower proportions throughout the study (Figure 2A).

**Figure 3.**
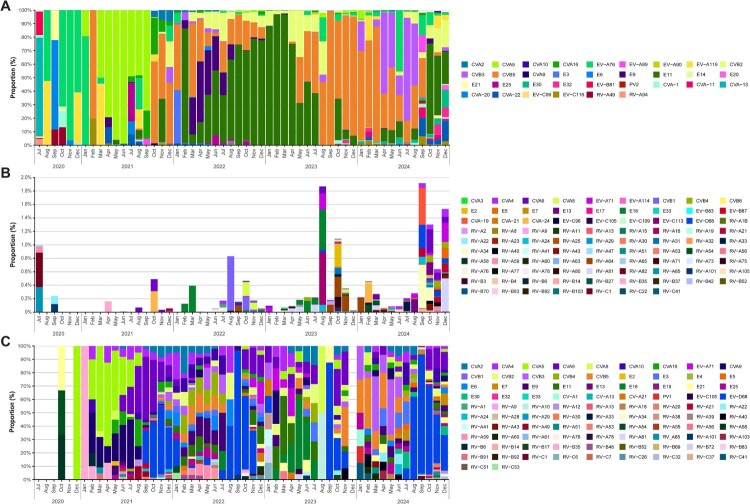
Relative abundance of EV types in Barcelona during 2020–2024. (A) Monthly characterization of EV types found in sewage with an abundance >1% and (B) with an abundance <1%. Each bar represents the mean percentage from both Barcelona WWTPs (PDL and BSS). (C) Monthly percentage of EV types found in clinical cases associated with medical assistance.

A total of 122 different EV types were found in sewage. Of these, 34 were detected in proportions >1% (Figure 3A) and 88 were found in proportions <1% (Figure 3B). Establishing a threshold of dominance of 40%, different EV types were found to be dominant throughout the years. EV-A76 was predominant from Oct-2020 to Jan-2021, CVA5 from Mar-2021 to Sep-2021, E11 from mid-2022 to mid-2023 and CVB5 from mid-2023 to mid-2024. The latter half of 2024 was mostly dominated by CVB3 and E11 (Figure 3A). Of note, cVDPV-2 was detected in Sep-Oct-2024 in the BSS WWTP, as well as in other regions of Europe in the same timeframe [7]. This event coincided with an upsurge of EV-C in the last months of 2024 (Figure 2A), where a high diversity of these species was detected in proportions higher than usual, especially CVA11, CVA22 and EV-C116 (Figure 3A).

### EV diversity in Barcelona clinical data

A total of 1.55 × 10^3^ sequences were obtained from clinical samples during the period of the study. All seven human-infecting EV species were found in clinical sequences, EV-A and B being the most abundant and EV-C and RV-C being the less prevalent. A seasonal trend was observed, with EV-A and B dominating from late-winter to mid-summer and EV-D (EV-D68) appearing from early-autumn to mid-winter (Figure 2B).

A total of 92 EV types were detected showing a wide monthly variety (Figure 3C). Only on very few occasions was there a dominating type. EV-D68 was the most common dominating type (29%) throughout the study (in Sep-Oct-2022, Sep-2023, and between Aug-Oct-2024), pointing to a high relevance of EV-D68 in clinical data. Although not always reaching the established threshold of dominance, the top EV types found during this surveillance were CVA6 (13%), CVA4 (4%), CVA9 (4%), CVB3 (4%), CVB5 (4%), CVA5 (4%), CVA2 (3%), CVA10 (3%), CVA16 (3%), E9 (3%), E11 (3%) and E18 (2%).

Of special attention is the report of a Sabin-like PV-1 infection detected in respiratory secretions of an immunosuppressed 2-year-old child in Jan-2024 (Figure 3C). However, detection of the virus was unsuccessful in the patient’s stool samples and in sewer samples taken in the vicinity of the patient’s home during the time of the infection. No paralysis-related symptomatology was reported.

### Correlation analysis of EV type abundance in clinical and sewage surveillance

To assess the potential of wastewater as a sentinel system for EV surveillance for specific EV types, correlation analysis between aggregated clinical cases two weeks after the sewage sampling date and their GC levels in wastewater for the years 2023 and 2024 was performed, as no pandemic-related measures were active. Fifteen EV types particularly abundant (≥40%) in wastewater or in clinical data were selected, also including EV-A71 due to its clinical relevance. CVA4, CVA6, CVB2, CVB3, E11 and EV-D68 were the ones showing the strongest correlations (Table 1). Although EV-A71 is typically transmitted via the faecal-oral route [24], no correlation was found for this type. No correlation was found for CVA2, CVA5, CVA16, CVA9, CVB4, CVB5, E6, and E9. The quadratic fit showed poor *R*^2^ values (*R*^2^ = 0.01–0.29) for all EV types except for CVA4, CVA6 and EV-D68 which showed fair correlations (*R*^2^ = 0.46, 0.61 and 0.62, respectively) (Table 1). However, model prediction error (0.21–9.07 cases) approached the total observed case range in most cases. CVA4 and CVB4 showed significant autocorrelation, whereas the other detected EV types did not. Taken altogether, these results indicate limited robustness in clinical case prediction for all studied EV types.

**Table 1.** Correlation and statistical analysis between EV type genome copies (GC) in sewage and 14-day accumulated cases after wastewater sampling.

Enterovirus type	ρ with aggregated cases 2 weeks after	Quadratic *R*^2^	Std. prediction error (cases)	Box-Ljung Χ^2^
CVA2	0.306	0.06	0.58	7.44
CVA4	0.55**	0.46	0.43	35.74***
CVA5	0.14	0.19	0.36	11.02
CVA6	0.611*	0.61	1.05	6.76
CVA16	0.11	0.26	0.34	11.83
EV-A71	0.055	0.04	0.43	4.27
CVA9	0.197	0.08	0.51	20.24
CVB2	0.574*	0.29	0.81	10.07
CVB3	0.47*	0.18	1.61	4.38
CVB4	−0.11	0.01	0.21	24.26*
CVB5	0.33	0.14	1.05	8.58
E6	0.11	0.15	0.27	0.58
E9	0.19	0.05	0.64	6.33
E11	0.45*	0.21	1.11	9.01
EV-D68	0.83***	0.62	9.07	2.51

ρ = Spearman’s rank correlation coefficient; Quadratic *R*^2^ = coefficient of determination from the quadratic regression model for wastewater GC; Standard prediction error = prediction error from the quadratic regression model expressed in number of cases; Box-Ljung Χ^2^ = test for temporal autocorrelation. * *p* < 0.05, ** *p* < 0.001, *** *p* < 0.0001.

### Phylogenetic relationships between EV in sewage and clinical isolates

**Figure 4.**
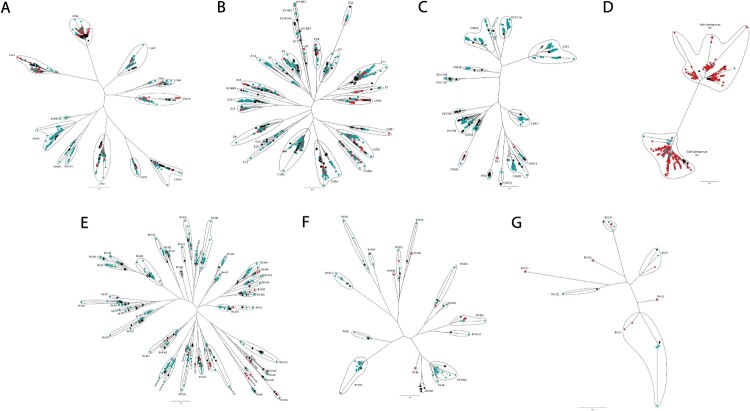
Phylogenetic analysis of EV found in sewage and clinical isolates. Phylogenetic trees for each individual EV species; (A) EV-A, (B) EV-B, (C) EV-C, (D) EV-D, (E) RV-A, (F) RV-B and (G) RV-C. Wastewater sequences are coloured blue, clinical sequences are coloured in red and reference sequences are coloured in black.

Phylogenetic analysis performed for each species showed a high intra-type genetic diversity, with circulation of multiple genetic clusters in the community (Figure 4A-G). While all EVs found both in sewage and at the hospital showed a close genetic relationship, 56 EV types were only detected in wastewater. Mean genetic identity between clusters corresponding to each EV type obtained from sewage samples and our generic EV database was of 88.1% (99.4%−80.0%), whereas mean genetic identity between clusters found in sewage samples compared to clinical sequences was 93.3% (100%–80.0%), indicating a close genetic relationship between environmental and clinical isolates in the area of study.

Despite sequencing a partial VP1 fragment, EV subtyping could be achieved for EV-A71 and EV-D68. For the former, only sub-genogroup C1 was detected throughout the study, whereas for the latter, sub-genogroups A2 and B3 could be detected (Figure 4D). However, the circulation of other subtypes cannot be ruled out as no full VP1 sequences were used in this study. Even at the sub-genogroup level, a high similarity between sewage and clinical sequences was observed.

The most abundant EV types were selected to identify amino acid mutations which occurred only in wastewater sequences and at frequencies over 10%. Six amino acid substitutions were detected. Specifically, VP1-K98R was found in 17.98% of wastewater CVA9 sequences, VP1-S87N in 19.73% of E9 sequences and VP1-I64L, VP1-V68L, VP1-F76Y and M110L were found in 87.72%, 87.72%, 75.99% and 87.74%, respectively, in CVB3 sequences. All these mutations had been previously reported in published sequences (NCBI). Few other mutations which have been previously reported in the literature to affect *in vitro* replication phenotype, antigenicity or virulence of certain strains [25–27] were also detected at lower proportions in environmental isolates only: VP1-T39 V in CVA9 (0.50%); VP1-P126L in CVB3 (1.73%) and VP1-D104N (1.46%) and VP1-C123Y (0.71%) in CVA16.

## Discussion

We present a 5-year EV surveillance study in the Barcelona metropolitan area combining clinical and wastewater analysis, with the aim of demonstrating the complementarity of both approaches during a particular period including the first years of the COVID-19 pandemic. Despite numerous factors that may influence the correlation between the number of EV clinical cases and their GC levels in sewage, both variables showed a similar seasonal pattern, particularly during 2023–2024. Spearman’s correlations were fair throughout the period of study, apart from 2022. Progressive withdrawal of pandemic-related measures during 2022 combined with a lower population immunity, may have resulted in a high number of infected individuals as proven by the high EV GC in sewage, albeit likely asymptomatic, which may partially explain the lack of correlation during that time. Lack of correlation in 2022 may also be partially explained by the specific distribution of EV types during that period, with an increasing predominance of E11 in wastewater (Figure 3A) which, as opposed to other EV types circulating during 2022, did not show a correlation with clinical cases (ρ = −0.31). E11 is expected to be found in wastewater at higher levels since it is principally transmitted via the faecal-oral route, showing higher levels of shedding in stool than in respiratory secretions [28,29]. Many other factors including the level and duration of faecal shedding of each type, environmental stability and association to clinical severity, may affect the correlation between EV environmental and clinical data.

Our study provided a comprehensive analysis of EV types circulating in the metropolitan area of Barcelona. Overall, 122 types of EV were found in sewage samples whereas 92 types were found in clinical isolates, and 66 different types were found both in sewage and clinical isolates. During 2020, only a limited number of clinical samples were detected and typed. The number of EV cases during that time was lower than usual, most likely due to reduced transmission associated with the implementation of pandemic control measures [30]. Many of the EV detected in sewage have been related to severe symptomatology such as AFP or AFM (EV-A71, CVA6, CVA9, CVB3, CVB4, CVB5, E6, E9, E11, E25, CVA24, EV-D68, EV-C99) [8,31–35], myopericarditis (CVA16, CVB2, CVB3, CVB5, E9) [32,36,37], encephalitis (CVB3, CVB5, E9, E11) [32,38,39], meningitis (CVA9, CVA10, CVB3, CVB5, E9, E18, E30, CVA22, CVA24) [32,40–43] and other milder syndromes such as HFMD (CVA5, CVA6, CVA10, CVA16, CVB3, CVB5) [44–47]. Until the end of 2020 in wastewater, EV-A76 emerged as the dominant type (Figure 3A). Although occasionally associated with AFP cases [48], this type has been rarely reported in clinical settings [49], suggesting a high rate of asymptomatic infections, in agreement with its detection in wastewater in several parts of the world [14–16]. In line with this, EV-A76 continued to be detected in sewage throughout the remainder of the study period, but it was not identified in any clinical sample. Dominant EV types in wastewater shifted over time highlighting the virus dynamics, CVA5 being predominant throughout 2021, E11 and CVB5 throughout 2022–2024 and CVB3 found specially in 2024 (Figure 3A). At the clinical level, CVA5 was also more abundant during early 2021, while E11 was more abundant in the first half of 2023, and CVB5 in the first half of 2024; CVB3 was detected throughout all the study period (Figure 3C). Notably, environmental surveillance also provided evidence of the circulation of rare EV such as EV-A89, EV-A90, E32, EV-B81, CVA22, EV-C105 and EV-C116, as well as a cVDPV-2 detected in Sep-24 (Figure 3A-B).

At the clinical level, the most prevalent type was EV-D68, mostly found from Sep-Dec 2021-2024, followed by CVA6, detected primarily from Jun-21 to Dec-21 (Figure 3C). Despite its abundance in the clinical setting, EV-D68 was only found in wastewater at very low proportions, likely due to its preferential shedding in respiratory secretions and/or a lower environmental stability. EV-A71, the principal cause of hand, foot and mouth disease (HFMD), although usually mild and self-limited, has also been related to other severe outcomes, such as in the outbreak of rhombencephalitis occurred in Catalonia during 2016 [50]. In our study, EV-A71 was found in low proportions in both sewage (mostly from Oct-2022 to Feb-2023 and Jul-2024 onwards) (Figure 3B) and clinical isolates (Figure 3C). Sequences found corresponded to subtype C1, the same variant that mainly caused the outbreak in 2016. EV-C were rarely detected in clinical isolates, accounting for a significant discrepancy with sewage data, where EV-C types, although typically found in small proportions, were usually detected. An underlying threat lies beneath detection of EV-C species, as EV are prone to recombination between types from the same species, and emergence of a new poliovirus-like virus could arise from recombination events between type C species [51,52]. Finally, despite the detection of RV from species A, B and C during the surveillance period with a great variety of types, the relative abundance of these species in sewage was also lower than 1% in most samples. We attribute the low presence of these species in sewage mainly to the fact that RV cause mostly respiratory diseases, as these viruses are less stable under acidic conditions and are rapidly inactivated at low pH, which prevents gastrointestinal transmission and distinguishes them from acid-resistant enteroviruses [53].

To our knowledge, no studies have previously assessed correlations between abundance in sewage and clinical cases for specific EV types. We selected 15 EV types based on their clinical importance or their high abundance in sewage to assess the correlation between the number of GC in sewage and the aggregated number of clinical cases from two weeks after the sewage sampling date, and the quadratic fit to evaluate its early warning capacity (Table 1). Our findings suggest that for certain EV (CVA6, CVB2, CVB3, E11, and EV-D68), wastewater surveillance could help anticipate clinical trends, as moderate to strong monotonic correlations were observed. However, the low R² values from the quadratic fit (except for CVA4, CVA6 and EV-D68) and high standard prediction errors indicate poor precision in predicting absolute clinical case numbers. Although CVA4 showed a fair correlation, significant autocorrelation undermines its reliability as an early warning indicator. Taken together, these results suggest that while wastewater data can track general trends in EV circulation, based on our dataset it lacks the accuracy needed for precise prediction of clinical case counts. Monotonic relationships were not observed for CVA2, CVA5, CVA16, EV-A71, CVA9, CVB4, CVB5, E6 and E9 (Table 1), suggesting a less consistent temporal relation between sewage data and clinical cases.

Phylogenetic analysis revealed a close genetic relationship between sequences from both sources (Figure 4). Sequences from different time periods that were assigned to the same EV type clustered together (data not shown), suggesting that there was not an extensive genetic divergence within the same EV types during the period of the study. Regarding EV-D68, sequences from two different clusters: sub-genogroups A2 and B3, the latter having been related to some acute flaccid myelitis (AFM) cases [54], although not in Barcelona during the study period, were detected (Figure 4D). Sequences from different years could be found in both clusters, pointing to their co-circulation (data not shown).

In this study, we also explored the potential of NGS-based wastewater monitoring as a tool for identifying viral amino acid mutations which could inform on the biology of these viruses or could show epidemiological relevance. For CVA9, CVB3 and E9 types, we detected a few mutations which were particularly abundant in wastewater sequences but were not found in clinical isolates. The VP1-K98R conservative mutation in CVA9 does not lie in any described antigenic or receptor binding site, thus an impact on virus immune scape or virulence is unlikely [55]. Contrarily, the VP1-S87N mutation found in E9 lies within the BC loop, which is known to play a role in pathogenicity and capsid stability, and is an important immunogenic site [56,57], and could potentially affect antibody binding. Four highly abundant mutations were found in CVB3 environmental sequences. While mutations VP1-I64L, VP1-V68L and VP1-M110L [58] are conservative and are not expected to have a significant effect, mutation VP1-F76Y lies near the BC loop [56] thus potentially affecting antibody binding. Moreover, the four mutations were found together in 76% of wastewater sequences. To our knowledge, only one viral isolate carrying these co-occurring mutations from an aseptic meningitis case in Tokyo (GenBank BFU31328) has been reported to date.

Mutations previously documented in the literature as contributing to alterations in viral phenotype were detected at low frequencies (0.50–1.73%) in wastewater samples. VP1-T39V mutation in CVA9 lies in the conserved PALTAVETGHT motif which is related to virus uncoating [25]. Mutations in VP1-126 residue have been related to a loss of cardio virulence in CVB3, thus the found VP1-P126L mutation may have an attenuating effect [26]. Two amino acid changes for CV-A16, one in the BC loop (VP1-D104N) and another in the EF loop (VP1-C123Y), were found suggesting a potential effect on antibody neutralization [27]. Altogether these results highlight the complementary information provided by NGS from wastewater samples to detect and monitor both high and low abundant mutations.

There are some methodological limitations in this study. First, different sampling strategies, grab vs composite, may have introduced a bias in the analysis. Nevertheless, as several studies have demonstrated that grab and composite samples yield comparable results [59,60], and given that correlation analysis of EV levels in both WWTPs was consistently strong throughout the study period, this potential bias is likely negligible. Additionally, EV persistence in wastewater has not recently been extensively studied, however, factors such as direct sunlight, heat and turbidity have been described as important factors affecting the behaviour of enteroviruses in estuarine and surface waters [61]. Therefore, persistence in wastewater may differ between EV types and could introduce a bias on type-specific detection. Finally, although the use of partial VP1 sequences impedes the detection of recombination events and limits the study of phylogenetic relationships. since the aim of the study was to assess the EV diversity, sensitivity was prioritized over whole genome information.

In summary, while clinical testing has proven effective for detecting acute symptomatic EV infections, it may benefit from WS to better monitor the circulation of these viruses in the population. This study provides an updated insight into EV circulation and the epidemiological scenario in the largest metropolitan area in Northern Spain during a 5-year period and demonstrates that combining clinical surveillance with WS offers a comprehensive and proactive approach. WS may contribute to correct the potential bias that clinical surveillance may suffer due to the occurrence of asymptomatic infections that can be undiagnosed, thus enabling early detection of emerging threats, supporting public health decision-making, and reinforcing the importance of environmental surveillance. Our findings suggest that EV types found in wastewater and those found in clinical specimens are related, which can be of special interest in areas where EV clinical surveillance is not implemented, and wastewater surveillance could be a useful approach to unveil the EV diversity. Regarding the current global emergency in the fight against PV eradication, WS based on amplicon next generation sequencing stands as a key element for advancing towards the ultimate goal of eradication.

## Supplementary Material

Supplementary Material.docx

Fig S1.tif

## Data Availability

All of the data generated or analysed during the study are included in the article. The datasets used and/or analysed during the present research project are available from the corresponding author upon reasonable request. Wastewater and clinical sequences are available at 10.5281/zenodo.17151017.
