# Supplementary material for: Unveiling the enterovirus diversity in Barcelona, Spain (2020–2024) through wastewater and clinical surveillance
Source: Emerg Microbes Infect. 2025 Nov 25;14(1):2589547. doi: 10.1080/22221751.2025.2589547 (PMC12667296; doi:10.1080/22221751.2025.2589547)
Supplement: Supplementary Material.docx [file TEMI_A_2589547_SM7743.docx]

**Supplementary Figure 1. Spearman’s correlation analysis between the mean GC in sewage and number of clinical cases**. Correlations between sewage levels and accumulated clinical cases from two weeks prior, one week prior, the same day, one week after and two weeks after the date of sampling. Statistical significance is indicated by asterisks: * < 0.05, ** < 0.001, *** < 0.0001.
