# Supplementary figures and images for: Unveiling the enterovirus diversity in Barcelona, Spain (2020–2024) through wastewater and clinical surveillance
Source: Emerg Microbes Infect. 2025 Nov 25;14(1):2589547. doi: 10.1080/22221751.2025.2589547 (PMC12667296; doi:10.1080/22221751.2025.2589547)

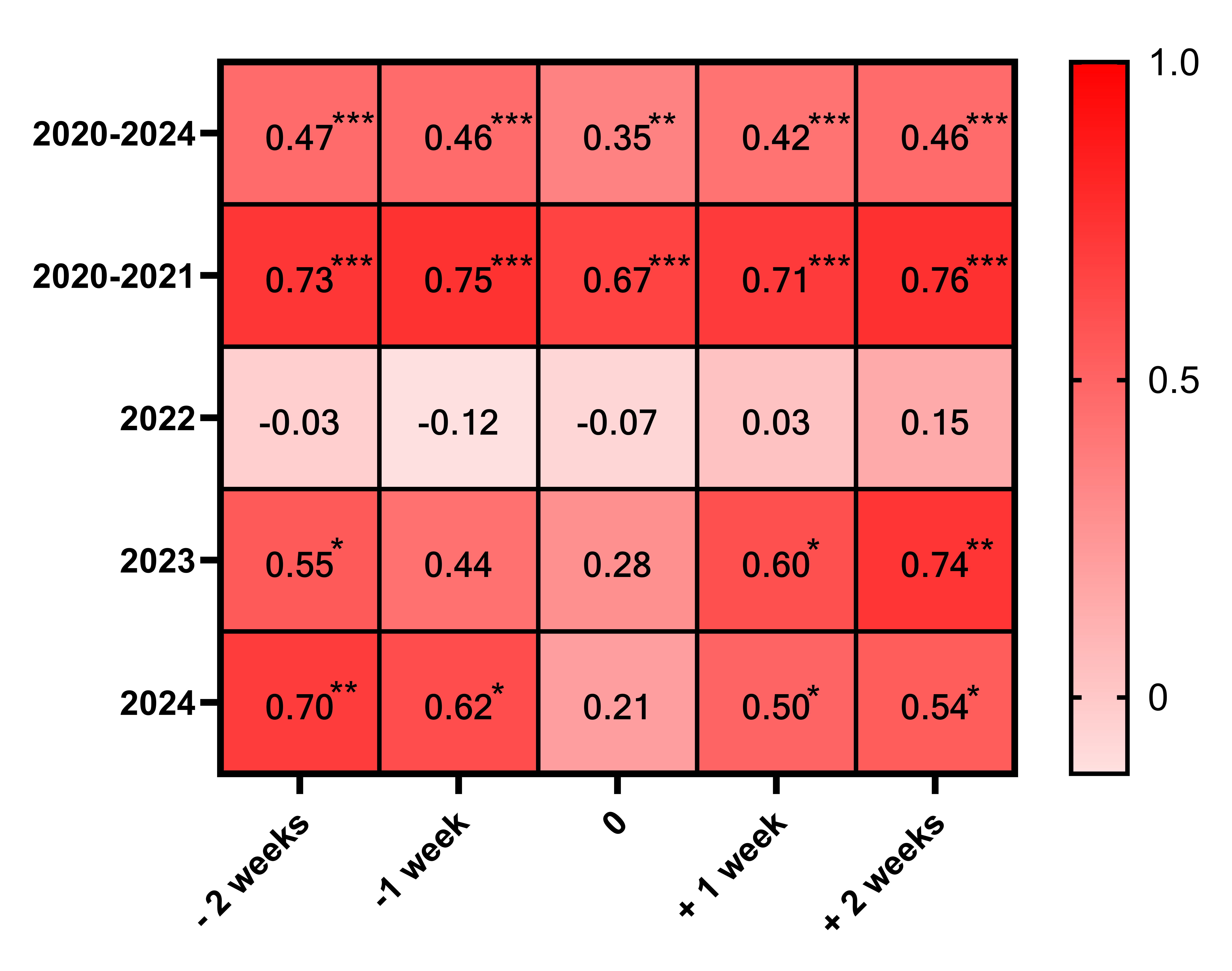

Supplement: Fig S1.tif [file TEMI_A_2589547_SM7742.tif]
